# Is Flexibility More than Fluency and Originality?

**DOI:** 10.3390/jintelligence10040096

**Published:** 2022-11-01

**Authors:** Selina Weiss, Oliver Wilhelm

**Affiliations:** Institute of Psychology and Pedagogy, Ulm University, Albert-Einstein Allee 47, 89081 Ulm, Germany

**Keywords:** creative flexibility, fluency, originality, working memory capacity, mental speed

## Abstract

Flexibility (i.e., the number of categorically different ideas), fluency (i.e., the answer quantity), and originality (i.e., the quality of ideas) are essential aspects of the ability to think divergently. Theoretically, fluency and ideational flexibility tasks are akin to one another. However, flexibility was also considered to be uniquely related to working memory capacity due to the task requirements involved in generating diverse answers (e.g., self-monitoring, suppression, and category generation). Given that the role of working memory is strengthened in flexibility tasks relative to fluency and originality tasks, flexibility should be more strongly related with working memory. Additionally, mental speed should show a similar pattern of results because mental speed has been previously related to task complexity. Based on a sample of *N* = 409 adults (M_age_ = 24.01 years), we found in latent variable models that fluency/originality strongly predicts flexibility and accounts for 61% of its variance. Creative flexibility was unrelated to working memory and mental speed after controlling for fluency/originality. Additionally, the residual of a latent flexibility factor was unrelated to self-reported creative activities. We concluded that flexibility, as measured here, can be deemed primarily a method factor that did not show value over and above fluency/originality as assessed in traditional fluency and originality tasks. We discussed perspectives for disentangling trait and method variance in flexibility tasks.

## 1. Introduction

The established understanding of divergent thinking—one way to assess creative thinking—is based upon the fluency (i.e., the number of ideas), the originality (i.e., the quality of ideas), and the flexibility (i.e., the diversity or variety of ideas) in idea production (e.g., Guilford 1956, 1967; Reiter-Palmon et al. 2019; Weiss et al. 2021b). Elaboration as a further aspect is rarely studied (Hornberg and Reiter-Palmon 2017). These aspects of divergent thinking can be studied in terms of latent constructs as they are not directly observable but inferred from variables that are observable (e.g., the quantity of answers regarding an alternate use item). In this manuscript, we therefore understood fluency, originality, and flexibility as latent variables that depict the prominent aspects of divergent thinking and have been linked with a number of ability and personality covariates as well as with creative outcomes (e.g., Batey and Furnham 2006; Benedek et al. 2012b; Feist 1998; Weiss et al. 2021a).

### 1.1. Understanding Flexibility

Flexibility is a cognitive ability that helps humans to achieve flexible behavior in different environments (Ionescu 2012). Flexibility has been defined based on different ideas (see Ionescu 2012): (a) as the cognitive ability to adapt to changing demands (e.g., task set-switching (Colzato et al. 2009; Monsell 2003)), (b) as a combination of flexibility and persistence (Nijstad et al. 2010), or (c) as one property of the cognitive system (e.g., modification of processes based on a change in task demands (Deák 2004)). Ionescu (2012) unified these views and ideas by describing flexibility as a general characteristic that is based on the interaction of knowledge, attention, monitoring, and executive functions with environmental cues such as task demands. When studied as an aspect of the ability to think divergently, flexibility is usually assessed in two instantiations: ideational flexibility and associative flexibility. Ideational flexibility describes the use of a variety of ideational categories (Guilford 1967; Runco 1986; Runco and Okuda 1991), whereas associative flexibility includes the ability to build a diversified association chain (Benedek et al. 2012b). However, in terms of understanding flexibility as an aspect of divergent thinking, it is predominantly conceptualized as ideational flexibility, and we will restrict our discussion to this aspect of flexibility.

Ideational flexibility is a prominent aspect in models of cognitive abilities (e.g., Carroll 1993) and features saliently in theories of creativity (e.g., Nijstad et al. 2010). Factor analytic studies of creative-thinking abilities (Wilson et al. 1954) list flexibility as a key aspect of idea production and creativity. It is described as the “adaptability to changing instructions”, the “freedom to inertia of thought”, and the “spontaneous shifting of sets” (Wilson et al. 1954, p. 298). In models of cognitive ability, flexibility is mostly described as an aspect of the general retrieval ability in the area of idea generation/production—such as in the Cattell-Horn-Carroll model (CHC, McGrew 2009). Within Carroll’s (1993) discussion of abilities in the domain of idea generation, several flexibility factors are listed in addition to fluency and originality factors. They date back to measures proposed in the Kit of Reference Tests for Cognitive Factors (Ekstrom et al. 1976; French et al. 1963) as summarized in Table 1. These factors all tap ideational flexibility and include the “shifting of sets” (Wilson et al. 1954) while differing between verbal and figural content.

The tests listed in Table 1 were all scored with regard to the number of spontaneous changes from one category to another (within the predefined categories (Carroll 1993)). The same scoring schemes of the predefined categories are still used in contemporary divergent thinking tasks that are instructed in a hybrid way for fluency as well as flexibility (e.g., Jäger et al. 1997; Reiter-Palmon et al. 2019) or specifically for flexibility (Runco and Okuda 1991). In fact, these tasks all seem inspired by the factor descriptions listed in Table 1 or the task materials published either by Wilson et al. (1954) or within the French Kits.

As described above, one idea for understanding flexibility is viewing it as a function of flexibility and persistence within a task. Both determinants are deemed essential for creativity and can mutually compensate each other. Originality can then emanate from the number of categories generated in a task but also from persisting in developing ideas within the categories in more depth (e.g., Nijstad et al. 2010, Zhang et al. 2020). Flexibility (and persistence) can therefore be understood as concepts that promote the generation of an original set of ideas (Nijstad et al. 2010). Flexibility is deemed a cognitive process that reflects the ease of subjects to switch to a different approach or different perspective. Persistence is defined as a process that reflects the degree of task-directed focused maximal cognitive effort and how this effort is sustained over time and distraction. Flexibility and persistence are seen as being substantially due to attention control and working memory—the former presumably being more strongly implied in the case of flexibility. Within models of divergent thinking, the flexibility of a person is usually assessed through the number of predefined categories used in a response. Persistence can be expressed as the mean number of solutions generated within a response category. Obviously, both persistence and flexibility are strongly related to fluency measures of the same task. In fact, multiplying the persistence with the flexibility score should deliver the sum of all solutions generated. In this sense, the dual pathway model is not a theory of creativity but a simple decomposition of fluency performance.

Previous factor analytical considerations have also strengthened the conclusion that meaningful individual differences can be equally found in fluency, originality, and flexibility (e.g., Carroll 1993; Wilson et al. 1954). However, the prominent usage of solely creative fluency indicators in the assessment of divergent thinking (Hargreaves and Bolton 1972) as well as the confounding effects of fluency in assessing originality and flexibility (e.g., Forthmann et al. 2020) appear to point to the direction that fluency is—in terms of reliability, validity, and scoring—a better indicator for capturing individual differences than originality or flexibility.

From a psychometric perspective, a key feature of flexibility is the approach to performance appraisals that stresses the diversity of responses. Evidently, unusual procedures—for instance, instructions that are incongruent with the performance appraisal—could be subject to explicit modelling. Evidently, fluency and originality are best understood as dispositional trait factors, whereas different methods of instructing subjects—for instance, performance appraisals that apply fluency scoring to an originality task—could be incorporated into overarching measurement models as so-called method factors (Eid et al. 2017; Weiss et al. 2021b). The strength of such method factors could provide us with an idea of how strongly such inconsistencies can affect the measurement of fluency and originality. Equally plausible, the instruction to provide persistent responses in a number of flexibility tests could also strengthen the relevance of some performance aspects relative to others.

In sum, ideational flexibility has been theoretically and empirically linked to divergent thinking (fluency and originality)—going back to several cognitive theories—but also to creative and original outcomes (Benedek et al. 2012b; Guilford 1967; Johnson-Laird 1988; Nijstad et al. 2010). Therefore, it is not surprising that flexibility and other aspects of divergent thinking—such as fluency and originality—are highly related. However, the correlations reported are so strong that the existence of flexibility beyond a less complex factor for fluency is questionable. In the literature, such high correlations with fluency have often led to understanding divergent thinking as unidimensional (e.g., Weiss et al. 2021a) or as evidence in favor of the so-called equal odds that describe the number of qualitative creative ideas as a linear function of fluency (e.g., Forthmann et al. 2020). Scores, such as those reported in Benedek et al. (2012a), that present a correlation on the manifest level of *r* = .86 between ideational fluency and ideational flexibility might be highly fluency confounded. Please note, that in Benedek and colleagues (and, unfortunately, many more studies) the tasks were scored so that fluency, originality, and flexibility were all assessed in any one task (Reiter-Palmon et al. 2019). Obviously, this causes stochastic dependencies that are at odds with standard psychometric models. More importantly, instructions to participants are inevitably opaque. Participants are often not aware if they should demonstrate fluency, originality, or flexibility. Often, they are simply instructed to be “creative” and are then coded for fluency, originality, and flexibility at the same time. Such unwanted stochastic dependencies can be avoided by instructing tasks only regarding one of these aspects of divergent thinking. Other studies replicated the strong relations between fluency and flexibility (e.g., *r* = .98, Shen et al. 2018) but also share this operational opaqueness. The key issue is that the instruction (e.g., be flexible) is mostly not congruent with the scoring (e.g., flexibility) (Reiter-Palmon et al. 2019). Therefore, published evidence can be deemed insufficient for estimating the amount of variance in flexibility measures that is accounted for by other factors such as originality and fluency. Other things being equal, more conclusive evidence should be based on tasks in which the scoring is congruent with the instruction given to participants.

Theoretically, fluency and ideational flexibility tasks are akin to one another. This means that, even though they capture different aspects of divergent thinking (quantity vs. diversity), they are theoretically related as diversity without quantity is not possible. It is also the case that a key feature—i.e., to produce a solution or an idea—is the same in both tasks, and instead of quickly producing a chain of conceptually similar responses, flexibility instructions stress diversity. Therefore, both concepts should be correlated and should show some uniqueness. In fact, once a categorization for responses is developed, fluency tasks can be instructed as flexibility tasks. Similarly, ideational flexibility tasks can be instructed as ordinary fluency tasks if the quantity of solutions is stressed and the diversity of responses is omitted from the instructions.

Besides, the ability to retrieve information from long-term storage is crucial for creative fluency and hence also for retrieving information from different categories in flexibility (Carroll 1993; Silvia et al. 2013; Unsworth et al. 2013). Verbal fluency and retrieval can be understood as hinging upon four ingredients (Rosen and Engle 1997). First, activation spreads in long-term memory starting with a cue delivered in the instruction. In flexibility tasks, more than one chain of activation might need to be pursued, thereby making such tasks harder, at least by virtue of putting a stronger load on cognitive control and working memory. Second, as a task progresses, more and more generated responses must be monitored to avoid repetitions and omissions. These executive functions are supposedly harder in tasks in which several chains of solutions are pursued, i.e., if solutions across a number of categories are generated instead of exhausting solutions from one category. Third, subjects will usually adhere to Grice’s maxims (Grice 1975) and avoid useless repetitions, although they are often not instructed as such. Therefore, generated solutions must be supervised, and this activity might put a higher strain on working memory if solutions are generated from different response categories. This should be the case because the response categories also need supervision in flexibility tasks apart from the solutions. The fourth ingredient is the self-generation of category cues to access new items (Rosen and Engle 1997). This includes that previously retrieved answers must be suppressed in order to access new answer categories. If this is not done, someone will resample already used categories and only generate answers within the same category.

Taken together, verbal fluency partly depends on working memory. In contrast, ideational flexibility should depend more strongly on working memory. Participants who are low in working memory should have more difficulties than those with high working memory in successfully monitoring and supervising solutions. They should also struggle to maintain response chains and the self-generation of cues. Given that the role of working memory is strengthened in flexibility tasks relative to fluency tasks, the difference between subjects with low and high working memory should be of higher magnitude in flexibility tasks. Similarly, mental speed should be more highly related with flexibility than with fluency. Flexibility tasks require more complex cognitive processes—maintaining response chains, etc.—that are not only demanding working memory capacity but also mental speed. Previous research has shown that general task complexity is related with mental speed and that this complexity is best understood as working memory requirements (e.g., Goecke et al. 2021; Sheppard and Vernon 2008). This implies that more complex tasks—as they require a greater amount of working memory—should show a higher correlation with mental speed than less complex tasks. An assumption based on that idea would be that flexibility, as the more complex task, is more strongly related with mental speed than fluency. However, on the contrary, Forthmann et al. (2019) have shown that general retrieval—which can be seen as the less complex task—is more strongly related with general mental speed than creative ideation (i.e., divergent thinking).

### 1.2. The Present Study

The two contrasting perspectives we wanted to present, therefore, allowed contrasting predictions. For example, suppose flexibility primarily constitutes a method factor. In that case, the relation between fluency and flexibility would be below unity, and the residual of a latent flexibility factor would show no meaningful relations with relevant covariates (working memory, mental speed, and creative activities). Similar ideas have been published in terms of the equal odds studying the confounding effects of fluency (Forthmann et al. 2020). Based on the ideas of equal odds, (Forthmann et al. 2020), a flexibility ratio score that is uncorrelated with fluency would fit the idea of such a method factor if both fluency and flexibility were scored on the same task. In the present paper, we used different tasks for assessing fluency and flexibility; therefore, the application of an equal odds scoring approach—despite its theoretical proximity to our ideas—could not be directly transferred to this analysis.

From the contrasting perspective, flexibility constitutes a lower-order factor of general retrieval ability just like fluency and originality, which all contain unique trait variance (Carroll 1993). As a result, the relationship between verbal fluency and flexibility should be below unity, and the residual of a latent flexibility factor should show meaningful relations with working memory. In order to overcome the obstacles of previous studies, we used instructions that were congruent with the scoring of all fluency, originality, and flexibility tasks. Based on structural equation modeling, we were able to test the above-described contrasting predictions. If flexibility is a unique construct, it is predicted by fluency/originality only to a limited degree. Based on the literature that reports correlations between flexibility and both working memory and mental speed, we would further assume that the flexibility factor shows significant relations with these constructs if it contains unique trait variance. Therefore, we first reported the correlations between fluency, originality, flexibility, working memory, and mental speed on a manifest level to replicate previous findings. Next, we went beyond previous research by testing if flexibility has unique trait variance beyond fluency/originality in a structural equation model.

## 2. Method

The following sections provide information on the study design, sample, and measures. The data and scripts that can be used to reproduce all analyses can be found online in OSF repository [https://osf.io/kh49m/].

### 2.1. Design and Participants

The current analysis was based on a larger study that included two studies conducted in three German cities (see Weiss et al. 2021a). The study was conducted in a computerized manner in computer laboratories. The lab session included—depending on the city—two to five hours of testing. All tasks used in this manuscript were administered in all cities. In Bamberg and Ulm, the test battery included five hours of testing and a variety of further covariates (e.g., the broad measurement of crystallized intelligence, insight tasks, personality, etc. (for a comprehensive list, see Weiss et al. 2021a)), while the test battery in Greifswald only included the creativity tasks and some intelligence indicators as this study was complemented by an EEG measurement (Kaur et al. 2020, 2021). The five-hour testing included various small and larger breaks to prevent fatigue. Additionally, the participants completed another two hours of online tests at home. The study at hand only included the data collected in the lab sessions. Included are various indicators for divergent thinking (fluency and originality (see Weiss et al. 2021a)), working memory capacity (Schmitz et al. 2018; Wilhelm et al. 2013), mental speed (Schmitz and Wilhelm 2016), flexibility (Schoppe 1975), and creative activities (Diedrich et al. 2018) that were used for the current analysis. Tests and questionnaires that were not subject to this study were not further described but can be found in Weiss et al. (2021a) and Goecke et al. (2020).

The participants were recruited through various channels (e.g., mailing lists and announcements in public places) and received monetary rewards for their participation in the study. The original sample included *N* = 457 participants. Due to missingness in the flexibility tasks, *N* = 33 participants were excluded from the analysis. The remaining sample *N* = 424 was cleaned regarding multivariate outliers. This procedure was performed using the Mahalanobis distance (see Meade and Craig 2012) which shows the standardized distance of a data point from the mean of the multivariate distribution. The Mahalanobis distance was calculated for multivariate outliers in flexibility, working memory, and mental speed. We excluded *n* = 15 outliers that showed a Mahalanobis distance > 15. The outliers were mostly low performers in the working memory and mental speed tasks who arguably had problems understanding the instructions.

The final sample used that was reported here included a total of *N* = 409 participants. A total of 31% of the participants were male, and the mean age of the sample was 24.01 years (ranging from 18 to 49 years (Hartshorne and Germine 2015)).

### 2.2. Measures

#### 2.2.1. Fluency and Originality

Fluency/originality as indicators of divergent thinking were assessed based on six tasks. Four tasks were indicators for fluency (including one figural fluency indicator), and two tasks were indicators for originality. The tasks were all open-ended and scored by two or three human raters (Reiter-Palmon et al. 2019). Descriptive statistics and intraclass correlations (ICC; Shrout and Fleiss 1979) can be found in Weiss et al. (2021a). The human rating included counting appropriate answers whenever the task was instructed for fluency (Reiter-Palmon et al. 2019). In the originality tasks, the human raters were trained to score each answer on a five-point Likert scale with regard to the uniqueness/uncommonness, remoteness, and cleverness of an answer (Silvia 2008; Silvia et al. 2009). Absent or inappropriate answers were coded as zero. Missing values in single tasks were due to computer problems and were deemed to be missing completely at random. The interrater reliability coefficients were mostly high, so aggregated scores across the human raters were used. In the following sections, the tasks measuring fluency and originality are described in more detail.

**Fluency.** All fluency tasks were open-ended and had a time limit. Participants were instructed to provide as many appropriate answers as possible within the given time. The similar attributes tasks (e.g., “Name as many things that you can that are ‘uneatable for humans’”) are similar to the alternate uses tasks and were based on items from the verbal creativity test (Schoppe 1975). Inventing names for abbreviations (e.g., “Invent names for the abbreviation: ‘T-E-F’”) was also adapted from Schoppe (1975). Another task was adapted and translated from the Kit of Reference for Cognitive Factors (Ekstrom et al. 1976). This task measured retrieval fluency (e.g., “Name as many household items”). Figural fluency comprised four paper–pencil tasks (e.g., “Draw as many objects as you can be based on a circle and a rectangle”) that were taken from the *Berliner Intelligenzstruktur-Test für Jugendliche: Begabungs- und Hochbegabungsdiagnostik* (Berlin Structure-of-Intelligence test for Youth: Diagnosis of Talents and Giftedness; Jäger 2006).

**Originality.** Originality was measured based on two open-ended indicators. In both tasks, the participants were instructed to provide a single answer that was very unique and original. Combining objects (e.g., “Combine two objects in order to build a door stopper in your house”) was adapted from the Kit of Reference Tests for Cognitive Factors (Ekstrom et al. 1976) and translated from English to the German language. The other task provoked the production of original nicknames (e.g., “Invent a combining objects for a bathtub”) and was adapted from (Schoppe 1975).

#### 2.2.2. Creative Flexibility

Flexibility was assessed based on six items from the four-word sentence task (Schoppe 1975). In this task, four letters were provided (e.g., T-G-F-U) that should be used to build a four-word sentence using words that begin with these letters (e.g., “Theo goes up furiously”/“Ulrike fears the goose”). The letters could be used in different orders, and no filler words were allowed. The participants were instructed to come up with as many different sentences as they could in a limited time period.

The task was coded for flexibility by three human raters. Flexibility can be scored by counting the number of predefined categories into which responses of a participant can be classified (Reiter-Palmon et al. 2019) or by counting the number of switches between such categories (Nusbaum and Silvia 2011). In the present study, we counted the number of categories used. Sentences were coded as flexible solutions if they fell semantically, syntactically, and/or in terms of content in different categories. Sentences from participants that were not classified into a novel category or sentences that did not adhere to the instructions (number of words, etc.) were coded as zero. Table 2 shows the items that were used along with the mean values of flexibility and the inter-rater reliability (Shrout and Fleiss 1979).

#### 2.2.3. Working Memory Capacity

Working memory was measured using a recall-1-back task (Schmitz et al. 2018; Wilhelm et al. 2013). These tasks were deployed using verbal stimuli (WMv) and figural stimuli (WMf). In the letter WMv task, the participants were presented with a letter in a box and were asked to type in that letter as soon as a new letter appeared. This was conducted with one to three boxes, implying that, with more boxes, the location of the previous letter had to be memorized next to remembering the current symbol. The tasks included a training phase with feedback and a test phase. In WMf, figures were displayed within a 3 × 3 matrix. Participants were instructed to indicate the figure that appeared last in the matrix at a given position while remembering the current stimulus. The task included a training phase with 21 trials and a test phase including 66 classifications. In both tasks, participants were, therefore, asked to identify the position where the same symbol occurred last while memorizing the current stimulus (see also Wilhelm et al. 2013).

#### 2.2.4. Mental Speed

The mental speed tasks included two verbal indicators and one figural indicator based on the comparison tasks (Schmitz and Wilhelm 2016). In these tasks, we presented two triplets of figures or letters simultaneously. The participants were instructed to decide if both triplets were identical. The task consisted of two blocks of 40 trials each. We used the reciprocal reaction time as an indicator within the measurement models. This score displays the correct answers per time.

#### 2.2.5. Inventory of Creative Activities and Achievement

The ICAA (Diedrich et al. 2018; Jauk et al. 2014) measures everyday creative activities in eight domains of creativity. The measurement includes the frequency of everyday creative activities based on the biographical assessment of the frequency of such behavior. As the ICAA’s long version showed problems regarding model fit, reliability, measurement invariance, and convergent validity, we used a short scale (S-ICA) that was compiled using the meta-heuristic ant colony optimization (Steger et al. 2022). The S-ICA included 8 items, one from each domain, and showed good model fit (CFI = .93; RMSEA = .05).

### 2.3. Statistical Analyses

#### Measurement Models

We computed several measurement models that were included in a larger structural model. For this, we used similar procedures to those described in Weiss et al. (2021a): for evaluating the fit of all models, we used the comparative fit index (CFI), the root mean square error of approximation (RMSEA), and the standardized root mean square residual (SRMR) (Hu and Bentler 1999). Applying these fit indices, CFI ≥ .95, RMSEA ≤ .06, and SRMR ≤ .08, indicated a very good fit. However, fit indices above CFI > .90 and RMSEA < .07 were acceptable. The statistical analysis was based on *r* software (version 3.6.2), using mostly the packages *lavaan* (Rosseel 2012) for all latent variable models and *psych* (Revelle 2018) for the outlier analysis and further descriptive statistics. All measurement models were estimated with the *maximum likelihood* (ML) estimator, and the structural model was estimated based on the *robust maximum likelihood* (MLR) estimator. We used the *full information ML* estimator to handle missing values (Schafer and Graham 2002). As a reliability estimate, we used McDonald’s ω (McDonald 1999; Raykov and Marcoulides 2011). The factor saturation (ω) for a factor indicates how much variance is accounted for by a latent variable in all underlying indicators (Brunner et al. 2012).

**Figure 1:**
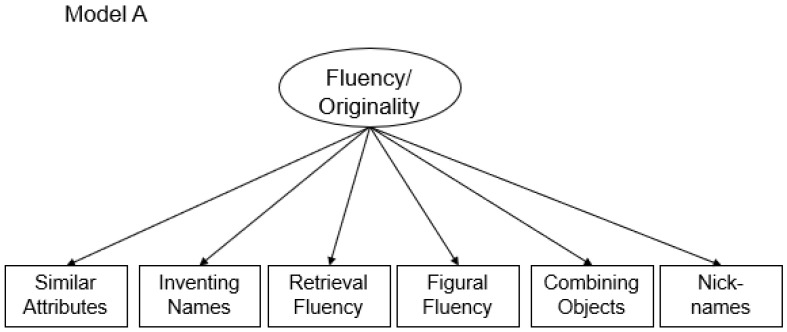

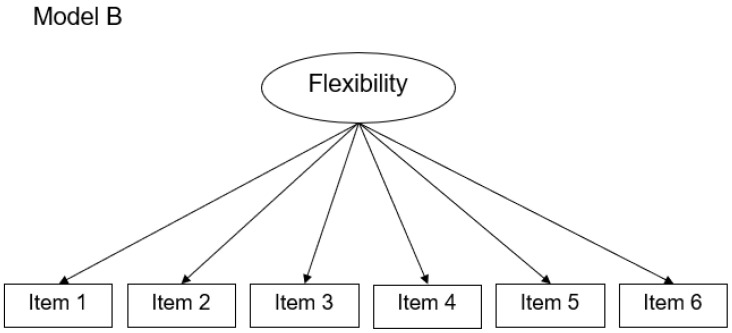
Schematic measurement model of fluency/originality (Model A) and flexibility (Model B).

Fluency and originality were modeled using a one-factor model as the latent correlation between fluency and originality was extremely high (*r* = .75), and a model including two correlated factors did not fit the data significantly better than a one-factor model (see Weiss et al. 2020; Weiss et al. 2021a). The previous studies using this data also showed that a separated originality factor failed to show significant variance. Therefore, to reduce the complexity of the structural model, we decided to stick to a one-factor model that captured fluency/originality in a single latent factor. This model fits the data well (χ^2^_(9)_ = 15.50; *p* = .08; CFI = .99; RMSEA = .04; and SRMR = .03). The reliability of the latent factor displaying fluency/originality was acceptable as ω = .74. Flexibility was modeled based on six items. The model fits the data well (χ^2^_(9)_ = 18.72; *p* = .03; CFI = .99; RMSEA = .05; and SRMR = .02) and showed good reliability as ω = .89. The factor loadings were all > .74. The model is schematically displayed in Figure 1.

## 3. Results

**Figure 2:**
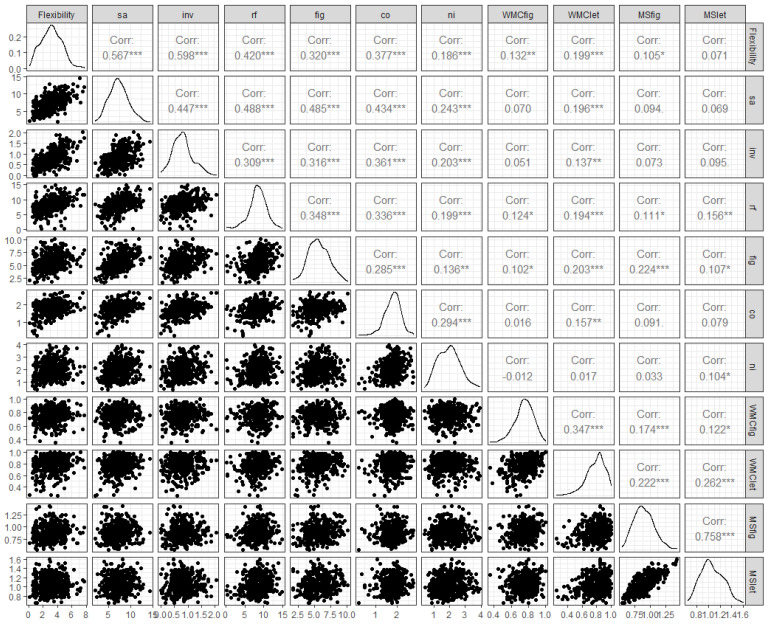
*Correlations on a manifest level: flexibility, fluency, originality, working memory, and mental speed.* Note: Statistically significant correlations are marked with * (*p* < .05), ** (*p* < .01), and *** (*p* < .001). Fluency indicators: sa = similar attributes; inv = inventing names; rf = retrieval fluency; and fig = figural fluency. Originality indicators: co = combining objects and ni = nicknames. WMC = working memory and MS = mental speed.

Based on the assumption that participants low in working memory should have more difficulties than subjects high in working memory in successfully monitoring and supervising solutions and that they should also struggle more with maintaining response chains and the self-generation of cues, we first analyzed the correlations between flexibility, working memory, and mental speed on a manifest level. In the first step, we correlated all variables on a manifest level. The results are presented in the scatterplot in Figure 2. Flexibility was significantly related with all fluency and originality indicators, although the correlations were higher with fluency (indicators: sa, inv, rf, and fig) than with originality (indicators: co and ni). Replicating previous results, flexibility also showed significant correlations with both working memory indicators and the indicator for figural mental speed. These relations were also salient in a confirmatory factor model that included flexibility, working memory, and mental speed (χ^2^_(34)_ = 47.37; *p* = .06; CFI = .99; and RMSEA = .03). Flexibility and mental speed were unrelated (*r* = .12), but working memory and flexibility did show a meaningful bivariate relation (*r* = .28).

In the next step, we estimated a larger model that included all of the variables in question. Here, flexibility was predicted by fluency/originality. To test if the variance left in flexibility was related with any of the covariates, we then correlated the residual of the latent flexibility factor with working memory, mental speed, and creative activities. As described above, if flexibility primarily constitutes a method factor, the relation between fluency and flexibility will be below unity, and the residual of a latent flexibility factor will show no meaningful relations with relevant covariates (working memory, mental speed, and creative activities). On the other hand, if flexibility constitutes a lower-order factor, it will contain unique trait variance. As a result, the relationship between verbal fluency and flexibility should be below unity, and the residual of a latent flexibility factor should show meaningful relations with working memory.

**Figure 3:**
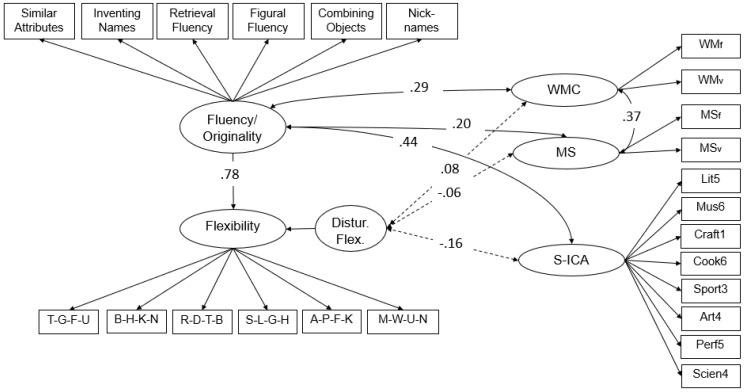
*Structural model: fluency/originality, flexibility, working memory, mental speed, and creative activities.* Note: S-ICA = short inventory of creative activities; Distur. Flex. = residual of latent flexibility; WM = working memory; and MS = mental speed. Non-significant correlations between S-ICA and WM/MS are not depicted.

This model (Figure 3) fits the data well: χ^2^_(244)_ = 423.84; *p* = .00; CFI = .93; RMSEA = .04; and SRMR = .06. Factor saturation was acceptable (ω_flu/org_ = .70, ω_Flex_ = .89, ω_S-ICA_ = .71, ω_WM_ = .49, and ω_MS_ = .86). Fluency/originality explained 61% of the variance in creative flexibility. As a further measure of creativity, the self-reported creative activities were not significantly related to working memory and mental speed but, as expected, were related with fluency/originality (*r* = .44). Fluency/originality, on the other side, was moderately correlated with mental speed (*r* = .20) and working memory (*r* = .29). The residual of the latent flexibility factor was not significantly related to any of the covariates (working memory, mental speed, or creative activities). Figure 3 displays the structural model, including these predictions and correlations.

A very similar pattern was found when the model presented in Figure 3 was estimated without the originality indicators: χ^2^_(201)_ = 348.80; *p* = .00; CFI = .94; RMSEA = .04; and SRMR = .06. In this case, a latent fluency factor predicted the flexibility factor slightly stronger (*β* = .81), while the residual of the latent flexibility factor was again not significantly related to any of the covariates (working memory, mental speed, or creative activities).

## 4. Discussion

Flexibility of thought is an important topic in creativity research. It has been described as a key ingredient of creativity (Nijstad et al. 2010), and it is theoretically akin to the fluency of ideas. However, flexibility has also been related with working memory capacity as it requires monitoring, suppression, and supervising responses (Rosen and Engle 1997). This raises the question of whether flexibility primarily constitutes a method factor or if it contains unique trait variance. In the next section, we summarized and interpreted our findings regarding flexibility.

### 4.1. Flexibility—A Method Factor

In previous studies, ideational flexibility has often been studied detached from other aspects of divergent thinking. For example, Benedek and colleagues found that ideational flexibility is significantly predicted by intelligence and inhibition (Benedek et al. 2012a). Their paper also assessed fluency, but they decided to reduce the model complexity by not modeling fluency and flexibility in one model as they were very highly correlated. Their finding underpins the theoretical idea that fluency and flexibility are akin to one another. Indeed, in many instances, fluency tasks can be turned into flexibility measures (and vice versa) by modifying instructions. It is also important that very strongly related variables are—ceteris paribus—unlikely to show divergent relations with other variables.

In the first step, we replicated previously found correlations on a manifest level and found that flexibility is related with divergent thinking, working memory, and mental speed. This finding suggested that participants low in working memory have more difficulties than subjects high in working memory in successfully monitoring and supervising solutions, and they should also struggle more with maintaining and updating previously generated responses and with the self-generation of cues to access new possible answers (Rosen and Engle 1997). Despite the initial assumption that mental speed is important in flexibility, because of the task complexity (Goecke et al. 2021), we did not find a significant relationship between flexibility and mental speed on a latent level. This finding converged with previous studies that report no or only small relations between mental speed and fluency/originality (e.g., Weiss et al. 2021a). On the other side, other studies report shared variance between mental speed and divergent thinking (Forthmann et al. 2019; Preckel et al. 2011). Indeed, the unspeeded relative to the speeded administration of divergent thinking tasks improved the quality of ideas (Forthmann et al. 2018). In the present data, fluency and flexibility tasks were speeded, but the originality tasks were unspeeded.

Given the theoretical proximity of fluency/originality and flexibility, previous results showing a high correlation between them might be misleading as fluency/originality are often not controlled for (e.g., Benedek et al. 2012a). Therefore, we next tested if flexibility can be subsumed below fluency/originality. The results showed that flexibility is strongly but not perfectly predicted by fluency/originality. This finding matched previous results (Benedek et al. 2012a). In an extended model, we were able to show that the residual of a latent flexibility factor shows no meaningful relations with working memory, mental speed, and creative activities. Given that flexibility could be subsumed below a more general fluency/originality factor and that its specificity was unrelated to previously suggested covariates, we suggested that the most adequate interpretation of flexibility is that it represents one out of a number of task classes of divergent thinking that did not show a meaningful specificity in the present study. Our findings, therefore, suggested that ideational flexibility is best understood as a method factor with no incremental validity.

### 4.2. Future Directions of Research

The findings implied that the aspects of divergent thinking—as discussed and measured hitherto—might need some revision. Creative fluency is the most prominent factor in previous research and this manuscript, and previous research shows that originality and flexibility are of limited utility over and above fluency (Hargreaves and Bolton 1972). However, a number of articles have provided evidence for the theoretical and practical importance of originality and flexibility, and based on novel automated scoring approaches (e.g., Reiter-Palmon et al. 2019) or the adaption of older scoring approaches (e.g., Forthmann and Doebler 2022), an incremental utility of originality and flexibility is conceivable. This implies that either the tasks or associated performance appraisal standards must be improved in order to better capture unique variance of flexibility and originality. Alternatively, the concept of divergent thinking should be revised towards a construct that is best depicted based on indicators of fluency. Such a revision would imply losing theoretically important aspects of *creativity*, such as the creation of novel ideas, and would therefore narrow the construct to retrieving previously learned information. Prior to discarding flexibility from divergent thinking, we propose a number of interventions to strengthen the flexibility measurement.

Even though this multivariate study included a variety of indicators that assessed fluency and originality, the indicator that was used to assess flexibility was limited to a single task with six items (four-word sentences; Schoppe 1975). This limited the findings as the employment of a single paradigm exacerbates the distinction between trait and method variance. Methodological specificities of this ideational flexibility paradigm might be crucial for the results reported. Therefore, administering additional flexibility measures, preferably also including associational flexibility (Benedek et al. 2012b), the flexibility of use, and indicators for figural flexibility (Ekstrom et al. 1976; French et al. 1963) in a replication and extension of the present results, would deliver more conclusive results concerning the utility of flexibility. Studying a broader range of flexibility tasks would allow the testing of whether or not flexibility is best understood as a flexibility method factor below an overarching fluency/originality factor. If we pursue such research, we should be open to the result that the diversity of responses (usually collected in measures that could serve as fluency tasks if instructed as such) is not essential for capturing individual differences in divergent thinking beyond fluency/originality. In this case, it would be better to focus on originality as that facet seems to build the true core of creativity, but it often has limitations in terms of measurement. The conclusion that flexibility does not capture variance beyond fluency/originality does not necessarily contradict theories such as the dual pathway theory (Nijstad et al. 2010). This model could—for instance—be further addressed by assessing if flexibility mediates the relationship between fluency and originality. On the other side, we should be open to the perspective that the concept of flexibility is mostly reflecting increased demands on attention control and working memory rather than performance aspects that should be deemed intrinsic to creativity. If that were the case, dismissing flexibility from the realm of creativity would be the correct conclusion.

According to Ockham’s razor, flexibility tasks need to capture something relevant that goes beyond what is measured with fluency tasks; if not, it makes sense to stick to fluency tasks because they are the more basic concept, and they are cheaper to administer and score (Hargreaves and Bolton 1972). Costs of scoring are usually substantial, given that all responses in flexibility tasks require human attention. Studies that capitalize on technological developments to evaluate responses from flexibility tasks could greatly facilitate and thereby cut the cost of scoring flexibility tasks. One such technological approach builds upon a semantic analysis of answers (Cosgrove et al. 2021). In the semantic analysis, the participants’ responses can be used to generate semantic networks displaying the connectivity of various nodes (words) in the semantic space (Cosgrove et al. 2021; Li et al. 2021). Such lexical network structures can categorize answers and express flexibility based on different indices, such as clustering coefficients, average path lengths between clusters and words, network modularity, and network diameter (Boccaletti et al. 2006; Kenett et al. 2016). Similar approaches are imaginable for processing images by blending images or comparing various image vectors and properties with one another (Rafner et al. 2020).

The flexibility within lexical network structures was mostly restricted to single words in the last years. Additionally, most of these studies use fluency tasks (that are instructed for fluency), but they are later scored for the semantic flexibility of participants (Kenett et al. 2016). This implies that flexibility is assessed based on the responses given to a fluency task, which is incongruent with the instruction (Reiter-Palmon et al. 2019). Studying semantic networks in flexibility tasks with congruent instructions (i.e., participants are instructed to maximize the diversity of their responses) would be of great interest. In addition, most semantic network approaches calculate networks for different groups (e.g., low creativity and high creativity (Li et al. 2021) or low intelligence and high intelligence (Kenett et al. 2016)). Such a procedure requires an a priori set threshold that allows for splitting the sample into low and high ability groups. This comes along with a loss of information. Therefore, future lexical network studies should focus on dimensional approaches. For instance, flexibility networks could be established along the working memory continuum using locally weighted structural equation models (Hildebrandt et al. 2016).

### 4.3. Conclusions and Outlook

The central question of this manuscript was to assess if flexibility primarily constitutes a method factor or if it contains unique trait variance. Our study showed that the residual of a latent flexibility factor is not meaningfully predicted by working memory, mental speed, and creative activities. This was in line with the idea that flexibility is best understood as one out of a number of divergent thinking concepts and that its specificity is not predictively useful (i.e., it is a method factor). As measured in this study, flexibility had no incremental validity above and beyond the broad assessment of fluency and originality. Therefore, flexibility mostly appeared as a twist to the more prominent fluency retrieval tasks but as a twist with an increased price tag attached to its use. Future research could either replicate and extend the present findings or could use technological tools to facilitate economic use of flexibility measures. We see potential in the analysis of semantic flexibility that captures a variety of semantic indices. Such an analysis should allow for dimensional considerations of variables (such as intelligence). Within such a novel approach, performance indicators, such as clustering coefficients, average path lengths between clusters and words, and network modularity, might be uniquely predicted by working memory and mental speed. Such indices have the potential to capture the supervision of generated answers, the suppression of previously used categories, and the activation of different category chains that might not all be perfectly captured in the traditional human count data.

## Figures and Tables

**Table 1:** Flexibility factors from the Kit of Reference Tests for Cognitive Factors (Ekstrom et al. 1976; French et al. 1963).

Factor	Description
Figural Adaptive Flexibility	“The ability to change set in order to meet new requirements imposed by figural problems” (p. 49)
Semantic Spontaneous Flexibility	“The ability to produce a diversity of verbally expressed ideas in a situation that is relatively unrestricted” (p. 50)
Figural Flexibility	“The ability to change set in order to generate new and different solutions to figural problems” (p. 73)
Flexibility of Use	“The mental set necessary to think of different uses for objects” (p. 197)

**Table 2:** Descriptive statistics for flexibility indicators, including ICCs.

Item	*Mean* (*SD*)	Min	Max	ICC
#1 (T-G-F-U)	3.08 (1.69)	0	8	.99
#2 (B-H-K-N)	3.72 (1.96)	0	10	.99
#3 (R-D-T-B)	2.87 (1.73)	0	9.67	.99
#4 (S-L-G-H)	3.47 (1.84)	0	10	.98
#5 (A-P-F-K)	2.82 (1.63)	0	8	.99
#6 (M-W-U-N)	2.97 (1.73)	0	8.33	.99

## Data Availability

We reported all data exclusions. The decisions surrounding final sample sizes were presented in the sample sections. The research objectives and hypothesis were not preregistered. The data and all required scripts are available at https://osf.io/kh49m/.
